# How Current Clinical Practice Guidelines for Low Back Pain Reflect Traditional Medicine in East Asian Countries: A Systematic Review of Clinical Practice Guidelines and Systematic Reviews

**DOI:** 10.1371/journal.pone.0088027

**Published:** 2014-02-05

**Authors:** Hyun-Woo Cho, Eui-Hyoung Hwang, Byungmook Lim, Kwang-Ho Heo, Jian-Ping Liu, Kiichiro Tsutani, Myeong Soo Lee, Byung-Cheul Shin

**Affiliations:** 1 Department of Rehabilitation Medicine of Korean Medicine, Spine and Joint Center, Pusan National University Korean Medicine Hospital, Yangsan, Republic of Korea; 2 Division of Clinical Medicine, School of Korean Medicine, Pusan National University, Yangsan, Republic of Korea; 3 Division of Humanities and Social Medicine, School of Korean Medicine, Pusan National University, Yangsan, Republic of Korea; 4 Center for Evidence-Based Chinese Medicine, Beijing University of Chinese Medicine, Beijing, China; 5 Department of Drug Policy and Management, Graduate School of Pharmaceutical Sciences, The University of Tokyo, Tokyo, Japan; 6 Brain Disease Research Center, Korea Institute of Oriental Medicine, Daejeon, Republic of Korea; University Of São Paulo, Brazil

## Abstract

**Objectives:**

The aims of this study were to investigate whether there is a gap between evidence of traditional medicine (TM) interventions in East-Asian countries from the current Clinical Practice Guidelines (CPGs) and evidence from current systematic reviews and meta-analyses (SR-MAs) and to analyze the impact of this gap on present CPGs.

**Methods:**

We examined 5 representative TM interventions in the health care systems of East-Asian countries. We searched seven relevant databases for CPGs to identify whether core CPGs included evidence of TM interventions, and we searched 11 databases for SR-MAs to re-evaluate current evidence on TM interventions. We then compared the gap between the evidence from CPGs and SR-MAs.

**Results:**

Thirteen CPGs and 22 SR-MAs met our inclusion criteria. Of the 13 CPGs, 7 CPGs (54%) mentioned TM interventions, and all were for acupuncture (only one was for both acupuncture and acupressure). However, the CPGs did not recommend acupuncture (or acupressure). Of 22 SR-MAs, 16 were for acupuncture, 5 for manual therapy, 1 for cupping, and none for moxibustion and herbal medicine. Comparing the evidence from CPGs and SR-MAs, an underestimation or omission of evidence for acupuncture, cupping, and manual therapy in current CPGs was detected. Thus, applying the results from the SR-MAs, we moderately recommend acupuncture for chronic LBP, but we inconclusively recommend acupuncture for (sub)acute LBP due to the limited current evidence. Furthermore, we weakly recommend cupping and manual therapy for both (sub)acute and chronic LBP. We cannot provide recommendations for moxibustion and herbal medicine due to a lack of evidence.

**Conclusions:**

The current CPGs did not fully reflect the evidence for TM interventions. As relevant studies such as SR-MAs are conducted and evidence increases, the current evidence on acupuncture, cupping, and manual therapy should be rigorously considered in the process of developing or updating the CPG system.

## Introduction

Low back pain (LBP) is a common condition that affects a significant proportion of the population, with an estimated prevalence of 70%–85% [1]. Current Clinical Practice Guidelines (CPGs) recommend various LBP treatments, such as pharmacotherapy, physical therapy, manual therapy, educational therapy, psychological therapy, and invasive therapy [2], [3].

Traditional medicine (TM) is defined as indigenous medicine used to maintain health and to prevent, diagnose, and treat physical and mental illnesses and is distinct from allopathic medicine based on theories, beliefs, and experiences [4]. In East-Asian countries, especially China, Korea, and Japan, the main therapeutic methods of TM consist of acupuncture, moxibustion, cupping therapy, herbal medicines, and manual therapies (called Tuina in China, Chuna in Korea, and Shiatsu in Japan) [5]. In East-Asian countries, 80% of the population depends on TM for primary health care, and 70% to 80% of the population in many developed countries has used some form of alternative or complementary medicine (e.g., acupuncture) [4]. Although studies on the use of TM are increasing [6], [7], differences in medical circumstances, culture, or poor evidence in support of TM seem to complicate the inclusion of TM in CPGs.

CPGs are systematically developed to assist practitioners and patients in making decisions about appropriate healthcare in specific clinical circumstances [8]. In contrast with previous approaches that were often based on tradition or authority, modern CPGs are based on an examination of current evidence within the paradigm of evidence-based medicine [9]. SR-MAs are literature reviews focused on a research question that attempts to identify, appraise, select, and synthesize all high-quality research evidence relevant to that question. SR-MAs of high-quality randomized controlled trials (RCTs) are crucial for evidence-based medicine [10]. Although it seems easy to write an SR-MA, good SR-MAs take time, and they frequently encounter delays but do not update the literature review. The additional typical delays for peer review and publishing add extra time, and SR-MAs may be printed two to four years after the end of the information retrieval. Finally, most SR-MAs are published worldwide without an accompanying CPG [11].

The purpose of this review was to investigate whether there is a gap between evidence of traditional medicine (TM) interventions in East-Asian countries from the current Clinical Practice guideline (CPGs) and evidence from current systematic reviews and meta-analyses (SR-MAs) and to analyze the impact of this gap on present CPGs.

## Methods

### Data Sources and Searches

Two types of databases were searched according to their database content. The first database was a CPG-related database for LBP that was used to understand the current status of LBP management. The other database included systematic reviews or meta- analyses (SR-MAs) and was used to compare the current evidence to current CPGs. Following the core, standard, ideal search (CoSI) model [12], we searched the following electronic databases from database inception to December 2012.

Our CPG database searches were the core searches because representative databases were more highly recommended than ideal searches. The CPG databases included the National Guideline Clearinghouse (NGC), Guidelines International Network (G-I-N), National Institute for Health and Clinical Excellence (NICE), and Scottish Intercollegiate Guidelines Network (SIGN). Additionally, we searched 3 representative East-Asian countries’ databases: the Chinese National Knowledge Infrastructure (CNKI) for China, the Korean Medical Guideline Information (KoMGI) for Korea, and the Medical Information Network Distribution Service (MINDS) for Japan.

For SR-MAs, we conducted an ideal search because all relevant SR-MAs of LBP were needed for the TM area. We found TM in the following databases: The Cochrane Database of Systematic Review (CDSR), PubMed, MEDLINE, EMBASE, DH-DATA, AMED, Chinese databases (China Knowledge Resource Integrated Database, Wanfang database, and Chinese VIP information), a Korean database (Oriental Medicine Advanced Searching Integrated System), and a Japanese database (Japan Medical Abstracts Society).

Each East-Asian country’s CPG and SR-MA databases were searched by authors from their own county.

The search keywords for CPG were (back pain OR low back pain OR lumbago) in each CPG database mentioned above. The search keywords for SR-MAs were (acupuncture OR acup*) for acupuncture, (moxa OR moxibustion) for moxibustion, (cupping) for cupping therapy, (herbal medicine OR traditional Chinese medicine OR Chinese herbal medicine) for herbal medicine, (manual therapy OR manipulation OR massage OR Chinese massage OR Tuina OR Chuna OR Shiatsu) for manual therapy, (low back pain OR back pain OR lumbago) for LBP, and (systematic review OR meta analysis OR meta analyze) for SR-MAs in each language. These search terms were combined in the form of [(LBP) AND (TM interventions) AND (SR-MA)]. This search strategy was adjusted for each database.

In addition, the bibliographies of relevant CPGs and SR-MAs were manually searched. Gray literature, consisting of theses, dissertations, letters, government documents, research reports, conference proceedings, and abstracts, was searched to avoid publication bias. The reference section for each study was searched. Personal contacts were made with the original authors of the searched studies to identify any data that were potentially missing from the publications.

The title and abstract of searched articles were read by a single primary researcher (H-WC), who conducted the screening process. Articles that were not written in English were translated into Korean or English prior to screening. The articles for potential inclusion in our review were checked by 2 independent reviewers (H-WC, E-HH). After screening the titles and abstracts retrieved in our search, we excluded all articles that did not meet our pre-defined inclusion/exclusion criteria. Then, the full text of the articles for inclusion was carefully read. The final inclusion was determined by two independent reviewers (H-WC, E-HH), who used the matching method.

### Study Selection

#### Types of CPG and SR-MA

Current CPGs regarding the treatment of non-specific LBP, which were used universally and considered the standard, were evaluated. When we conducted the preliminary search, there were few CPGs not written in English, and they were from the Netherlands, Spain, Germany, France, Finland, and Brazil. The CPGs’ development dates were relatively older than the English versions, and the relevance of their content was low. Thus, we concluded that they would have no effect on the analysis. Because we wanted to show the current state of TM through the representative CPGs, the authors reached a consensus to limit the language of the CPGs to English.

Non-specific LBP was searched and evaluated to understand the current evidence from SR-MAs research studies on the effectiveness of 5 major TM interventions (acupuncture, moxibustion, cupping therapy, manual therapy, and herbal medicine). Language was not restricted during the selection of SR and MA.

#### Types of participants in CPG and SR-MA

LBP was defined as pain localized to the area between the costal margin or the 12th rib to the inferior gluteal fold. Non-specific LBP indicated the lack of a detectable specific cause, such as infection, neoplasm, metastasis, osteoporosis, rheumatoid arthritis, fracture, or inflammatory process [13].

The CPGs and SR-MAs in our review included all stages of non-specific LBP with or without radiating pain, such as acute (lasting up to six weeks), sub-acute (lasting six to 12 weeks), or chronic (lasting longer than 12 weeks) non-specific LBP [14].

#### Types of interventions in SR and MA

We analyze the TM of the primary therapeutic interventions, including acupuncture, moxibustion, cupping therapy, herbal medicines, and manual therapies, found in East-Asian countries. We selected these 5 types of interventions because they were medical insurance reimbursement items in East-Asian countries [15].

1. Acupuncture: only included needle acupuncture with or without electrical stimulation. Acupuncture without needling, such as laser or TENS on acupoints, was excluded.
2. Moxibustion: included when acupoints were heated with moxibustion.
3. Cupping therapy: included both dry and wet cupping.
4. Manual therapy: included Tuina in China or Chuna in Korea. Massage techniques were included, such as Chinese massage, acupressure, acupuncture massage, or Shiatsu when applied to acupoints or meridians.
5. Herbal medicine: included herbal medicine according to the TM diagnosis.

When studies addressed various symptoms or interventions in one acupuncture SR, we limited the inclusion criteria if the majority (>50%) of the participants and the intervention were acceptable for predefined criteria because there were numerous acupuncture SR-MAs. However, there were few available SR-MAs of moxibustion, cupping, manual therapy, and herbal medicine. Therefore, we included the SR-MAs when the RCTs of those interventions were greater than 10% of all RCTs when the data could be separately extracted.

When it was difficult to evaluate the independent effectiveness of TM intervention, such as comparing the same interventions or mixed treatments, the SR-MAs were excluded.

### Data Extraction and Quality Assessment

Two reviewers (H-WC, E-HH) independently extracted the data based on predefined characteristics to describe each study (refer to Tables 1, 2). In CPG, we extracted the type of interventions, the presence of TM, and the recommendation. In SR-MAs, we extracted outcome measures and their directions of outcome for each intervention and condition of LBP.

**Table 1:** Comparison of Clinical Practice Guidelines for Low Back Pain.

Database	Guideline & Year	Target population	Interventions and practices considered	Presence ofTraditionalMedicineInterventions	Recommendation	AGREEII OverallAssessment
NGC (USA)	NGC-8959/2012 [21]	(Sub)acute/Non-specific LBP with or withoutradiculopathy/ including pregnant women	1, 2, 3, 5	None	NA	6/Y
	NGC-8744/2011 [24]	(Sub)acute/Non-specific LBP with or withoutback-related legsymptoms	1, 2, 3	None	NA	5/YWM
	NGC-8517/2011 [28]	(Sub)acute & chronic/Non- specific LBP	1, 2, 3, 5, 6	Yes (acupuncture/Acupressure)	Acupuncture/Acupressureconsidered, but are notrecommended	6/Y
	NGC-8193/2010 [22]	(Sub)acute & chronic/Non- specific LBP with or without radiculopathy	1,2,3,4	None	NA	3/YWM
	NGC-8009/2010 [26]	(Sub)acute/Non-specific LBP	1,2,3,6	None	NA	3/YWM
	NGC-7704/2009 [25]	(Sub)acute & chronic/Non-specific LBP	(sub)acute LBP : 1, 2, 3, 5, 6/ Chronic LBP : 1, 2, 5, 6	Yes (acupuncture)	1. (Sub)acute: acupuncture -Do Not Know/2.Chronic: acupuncture - Do	3/YWM
	NGC-7510/2009 [27]	Work-related injuries or illnesses related tothe low back, elbow,shoulder, forearm, wrist,or hand	2, 3, 4	None	NA	3/YWM
	NGC-7428/2009 [2]	Chronic/Non-specific LBP	6	None	NA	4/YWM
	NGC-6456/2007 [23]	Work-related low backdisorders with radiculopath	1, 2, 3, 5, 6	Yes (acupuncture)	1.(Sub)acute LBP:acupuncture - Not recommended (Insufficient)/2.Chronic LBP: acupuncturefor select use during a limitedcourse with a clear objectiveand functional goals –Recommended (C-weak)/acupuncture - Notrecommended (Insufficient)	4/YWM
	NGC-5968/2007 [20]	(Sub)acute & chronic/Non- specific LBP	1, 2, 3, 4, 5, 6	Yes (acupuncture)	Moderate quality evidence,Weak recommendation	3/YWM
NICE (UK)	CG-88/2009* [29]	(Sub)acute & chronic/Non-specific LBP	1, 2, 3, 4, 5, 6	Yes (acupuncture)	Consider offering a courseof acupuncture needlingcomprising up to a maximum of 10 sessions over a periodof up to 12 weeks.	5/YWM
G-I-N(International)	Prodigy(UK) Backpain - low(withoutradiculopathy)/2009 [3]	(Sub)acute & chronic/Non-specific LBP withoutradiculopathy(sciatica)(including sprains and strains)	1, 2, 3, 4, 5, 6	Yes (acupuncture)	The course should have up to 10 sessions given over a period of up to 12 weeks	3/YWM
MINDS (Japan)	Clinical Practiceguideline for themanagement ofLBP/2012 [30]	(Sub)acute & chronic/Non-specific LBP	1, 2, 3, 4, 5, 6	Yes (acupuncture)	(Sub)acute – Do not know/Chronic- It is hard to sayacupuncture is better thanother conservative therapies.	4/YWM

Abbreviations: LBP, low back pain; AT, acupuncture; NA, Not applicable YMA, yes with modification; Y, yes.

Items of Interventions and practices: 1 = pharmacological therapy, 2 = physical therapy, 3 = education, 4 = psychological therapy, 5 = manual therapy, 6 = invasive therapy; Items of outcomes considered: 1 = pain, 2 = Global measure, 3 = functional status, 4 = Quality of Life, 5 = Safety, 6 = Cost effectiveness, 7 = Other outcomes.; All AGREE II items are rated with the following 7-point scale: Score of 1 (Strongly Disagree) = There is no information relevant to the AGREE II item or very poor reporting of the concept.; Score of 7 (Strongly Agree) = quality of reporting is exceptional and the full criteria and considerations articulated in the User’s Manual have been met.; Scores between 2 and 6 = The reporting of the AGREE II item does not meet the full criteria or considerations. A score is assigned depending on the completeness and quality of reporting. Scores increase as more criteria are met and considerations are addressed. We classified scores of 1 or 2 as low quality, scores of 3, 4 or 5 were moderate quality and 6 or 7 were high quality.; Domain scores are calculated by summing all the scores of the individual items in a domain and by scaling the total as a percentage of the maximum possible score for that domain. The scaled domain score will be: (Obtained score – Minimum possible) score/(Maximum possible score – Minimum possible score)*100.

*: NGC-7269 was originated from CG88 and it was summary of CG-88; thus, it was excluded.

**Table 2:** Systematic Reviews of Low Back Pain.

Type ofTraditionalMedicine	Stage ofLBP	FirstAuthor &Year	Intervention	Outcomemeasurement	Direction ofOutcome(Number of RCTs)	Level ofEvidence/Recommendation(SIGN)	Total AMSTA R score
Acupuncture							
	(Sub)acute						
		McIntosh 2008 [45]	Acupuncture	1, 2, 3, 7	P+(3)	1−/A	5
	Chronic						
		Hutchinson 2012 [44]	Acupuncture	1, 3, 4, 6, 7	P+ (7)	1+/A	4
		Trigkilidas 2010 [40]	Acupuncture	1, 3, 4, 6	I (4)	Not applicable	2
		Rubinstein 2010 [42]	Acupuncture	1, 3	P+ (18)	1+/A	10
		Yuan 2009 [39]	Acupuncture	1, 3, 4	Chronic LBP: P+ (23)	1+/A	7
		Ammendolia 2008 [37]	Acupuncture	1, 3, 4, 5, 7	I (19)	1+/A	4
		McIntosh 2008 [38]	Acupuncture	1, 2, 3, 7	P+ (32)	1−/A	4
		Henderson 2002 [34]	Acupuncture	Not reported	I (5)	2+/C	2
	Mixed						
		Furlan 2012 [41]	Acupuncture	1, 3, 5, 6	(Sub)acute LBP :I/chronic LBP : P+ (33)	1−/B	9
		Lu 2011 [43]	Acupuncture	1, 3, 4, 6, 7	P+ (5)	1+/A	8
		Furlan 2005 [35]	Acupuncture	1, 2, 3, 4, 5, 7	(Sub)acute LBP : I(3)/chronic LBP : P+ (32)	1+/A	3
		Maurits 2005 [46]	Acupuncture	1, 3, 7	acute LBP : I(2)/chronic LBP : P+ (13)	1+/A	9
		Manheimer 2005 [36]	Acupuncture	1, 2, 3, 7	P+ (33)	1+/B	8
		Ernst 2002 [33]	Acupuncture	1, 3	I (12)	1−/A	10
		Smith 2000 [32]	Acupuncture	1, 2, 7	(sub)acute LBP : N+(2)/chronic LBP : N+ (8)	1+/A	7
		Tulder 1999 [31]	Acupuncture	1, 3, 7	I (11)	1−/A	8
Cupping Therapy							
	Mixed						
		Kim 2011 [52]	Dry/Wet cupping	1, 5	(sub)acute &chronic LBP : P+ (2)	1−/B	8
Manual Therapy							
	Chronic						
		Kim 2012 [50]	Acupressure	1, 3, 7	P++ (3)	1−/B	10
		Imamura 2008 [47]	Acupuncture massage,Acupressure	1, 2, 3, 4, 5, 6, 7	P+ (4)	1+/A	5
	Mixed						
		Moon 2012 [51]	Chuna	1	P+ (2)	1−/B	6
		Robinson 2011 [49]	Shiatsu, Acupressure	1, 3	Shiatsu: I(1)/Acupressure : P+ (3)	1−/B	6
		Furlan 2009 [48]	Acupuncture massage,Acupressure	1, 2, 3, 4, 5, 6, 7	P++ (5)	1+/A	10

Abbreviations: LBP, low back pain; I, insufficient; P, positive; N, negative;

+ = weak; ++ = moderate; +++ = strong.

Items of outcomes measurement: 1 = pain; 2 = Global measure; 3 = functional status; 4 = Quality of Life; 5 = Safety; 6 = Cost effectiveness; 7 = Other outcomes.

The total AMSTAR score was calculated by adding the average scores for all 11 items. We averaged item scores across guidelines. Item scores were classified such that 0–3 indicated low quality, 4–7 indicated moderate quality and 8–11 indicated high quality.

#### Outcome measures

The outcome measures that we considered are described below. SR-MAs that used at least one outcome measure related to pain were included. The other outcome measures were considered, and their inclusion may be important for the study of LBP.

1. Primary outcome: Pain intensity
2. Secondary outcome: Global measure of improvement or recovery/Back-specific functional status/Quality of life/Safety/Cost-effectiveness/Other outcomes

### Level of Evidence and Recommendation

We reassessed the evidence level and recommendations of the SR-MAs using the SIGN grading system [16]. All disagreements were resolved through discussion and consensus or by the first author (H-WC).

### Quality Assessment of CPGs and SR-MAs

The SR-MAs of 24 different appraisal tools and some studies have shown that the Appraisal of Guidelines for Research & Evaluation (AGREE) instrument is an acceptable standard for guideline evaluation. Therefore, the AGREE Instrument for reporting the quality of CPGs was used [17], [18], and the Assessment of Multiple Systematic Reviews (AMSTAR) checklist for reporting the quality of SR-MAs was used to evaluate the methodological quality of the included publications. The AMSTAR instrument has recently been used in another study [19].

Four reviewers (H-WC, E-HH, K-HH, and B-CS) were fully trained in the quality assessment and data extraction methodology.

### Data Synthesis and Analysis

We identified the directions for future CPG of LBP through deep discussion and expert consensus among authors. All authors were CPG-related experts from East-Asian countries (China, Korea, and Japan). The authors discussed and reached consensus through e-mail contact. In cases of disagreement, the final recommendation was made by consensus. Per the authors’ recommendations, we recommend studies based on the results in Table 1 and Table 2.

## Results

### Study Description

**Figure 1:**
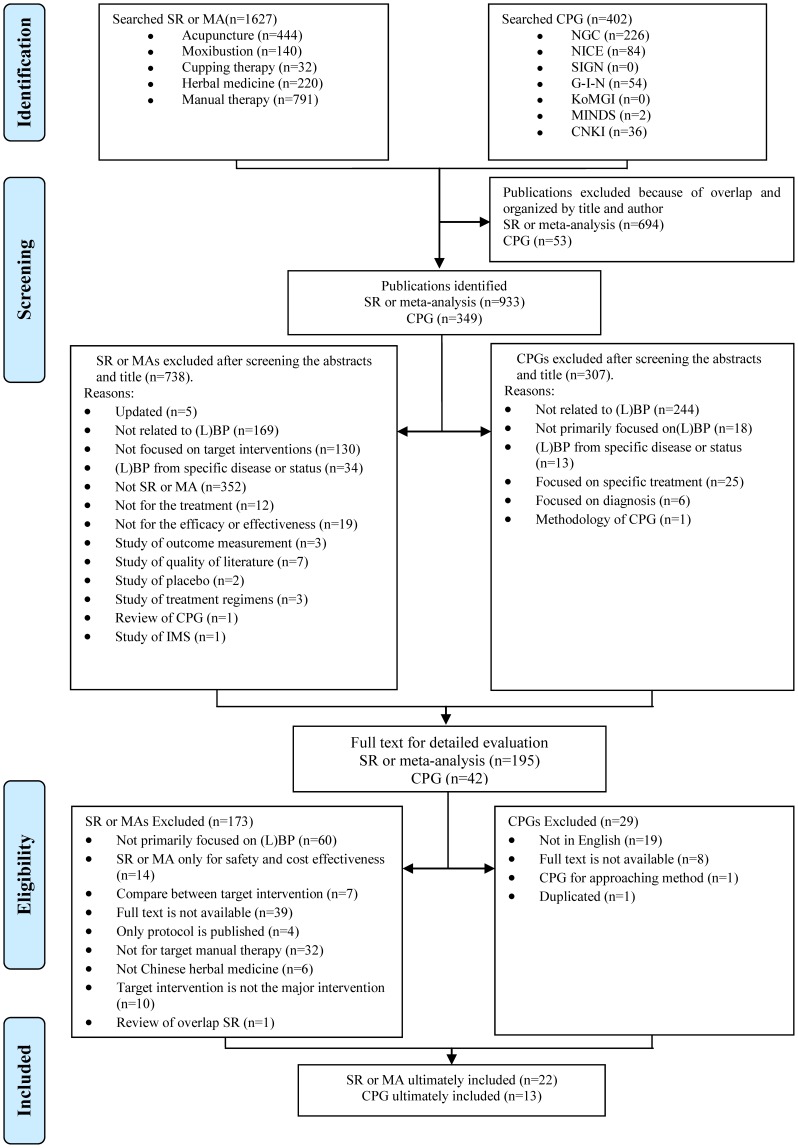
Flow chart of the study selection process. SR = systematic review; MA = meta-analysis; CPG = clinical practice guideline; (L)BP = (low) back pain; IMS = Intra Muscular Stimulation.

A total of 402 CPGs and 1627 SR-MAs were identified. After manually removing the duplicates and screening the titles and abstracts, 42 CPGs and 195 SR-MAs were identified as potentially relevant. After a detailed evaluation of the full text, 29 CPGs and 173 SR-MAs were excluded. Finally, 13 CPGs and 22 SR-MAs met our inclusion criteria. The literature search process is summarized in Figure 1, following the Preferred Reporting Items for Systematic Reviews and Meta-Analysis (PRISMA) flow diagram. The key data are summarized in Table 1.

### Current Clinical Practice Guidelines

Of the 13 CPGs, 10 originated in the USA [2], [20]–[28], 2 were from the UK [3], [29], and 1 was from Japan [30]. There were no CPGs from any East-Asian country. There were 7 CPGs for both (sub)acute and chronic LBP [3], [20], [22], [25], [28]–[30], 1 for chronic LBP [2], 3 for (sub)acute LBP [21], [24], [26], and 2 for work-related LBP [23], [27]. The CPGs addressed various interventions, such as pharmacological therapy, physical therapy, education, psychological therapy, manual therapy, and invasive therapy. However, TM interventions were only included in 7 CPGs [3], [20], [23], [25], [28]–[30]. All TM interventions were for acupuncture, and only 1 CPG [28] mentioned both acupuncture and acupressure.

Of 7 CPGs, 6 recommended acupuncture, but all of these CPGs had weak recommendation strength or made the recommendation with session limitations [3], [20], [23], [25], [29], [30]. However, 3 CPGs did not recommend acupuncture for (sub)acute LBP [23], [25], [28]. Only 1 CPG [28], which analyzed acupressure, did not recommend the treatment (Table 1).

### Quality Assessment of Clinical Practice Guidelines

The overall assessment mean of the included CPGs was 4±1 (range: 3–6), indicating that CPGs have moderate quality. There were 2 high-quality CPGs [21], [28], 11 moderate-quality CPGs [2], [3], [20], [22]–[27], [29], [30], and no low-quality CPGs. We assessed 2 CPGs [21], [28] as “recommend without modification” due to high quality and 11 other CPGs as “recommend with modifications” (Table 1). In each domain, the CPGs showed comparatively more than moderate quality (Table S1).

### Evidence of Systematic Review and Meta-analysis

Of 22 SR-MAs, 16 were for acupuncture [31]–[46], 5 were for manual therapy (Chuna, acupressure, acupuncture massage, shiatsu) [47]–[51], and 1 was for cupping therapy [52]. For chronic LBP, 1 SR reported a moderately negative conclusion [32], 5 SR-MAs reported an insufficient conclusion [31], [33], [34], [37], [40], and 9 SR-MAs reported weakly positive effects of acupuncture [35], [36], [38], [39], [41]–[44], [46]. For (sub)acute LBP, 1 SR reported a moderate negative conclusion [32], 5 SR-MAs reported insufficient conclusions [31], [33], [35], [41], [46], and 3 SR-MAs reported weakly positive effects of acupuncture [36], [38], [43]. The evidence level and recommendation strength were reassessed using the SIGN grading system. For the level of evidence, 5 SR-MAs were assessed 1− [31], [33], [38], [41], [45], 9 SR-MAs were 1+ [32], [35]–[37], [39], [42]–[44], [46], 1 SR was 2+ [34], and 1 SR was not applicable [40]. For recommendation strength, 12 SR-MAs were assessed as grade A [31]–[33], [35], [37]–[39], [42]–[46], 2 SR-MAs were grade B [36], [41], 1 was grade C [34],and 1 was not applicable [40].

Only 1 SR-MA on cupping for pain, including 2 RCTs for both (sub)acute and chronic LBP, reported weakly positive conclusions. The study partially considered pain and safety (adverse effect). The evidence level was assessed to be 1−, and the recommendation strength received a grade of B [52].

Of 5 SR-MAs of manual therapy, 1 considered Chuna [51], and 4 considered acupressure (including acupuncture massage and Shiatsu) [47]–[50]. All of these SR-MAs were compared with other interventions and reported positive conclusions, except for an inconclusive conclusion reported in 1 study of Shiatsu [49]. The study evidence level was assessed to be 1−, and the recommendation strength received a grade of B [51]. In 4 studies of acupressure, 2 were on chronic LBP alone [47], [50], and the other 2 included both (sub)acute LBP and chronic LBP [48], [49]. The evidence level was assessed to be 1− in 2 studies [49], [50] and 1+ in the other 2 studies [47], [48]. The recommendation strength received a grade of A in 2 studies [47], [48] and B in the other 2 studies [49], [50] (Table 2).

### Quality Assessment of Systematic Reviews

The mean (± standard deviation) score of total quality assessment of the SR-MAs was 6.59±2.65 (range: 2–10) (Table S2). A total of 10 SR-MAs (47.6%) were assessed to be high quality [31], [33], [36], [41]–[43], [46], [48], [50], [52], 9 SR-MAs (38.1%) were moderate quality [32], [37]–[39], [44], [45], [47], [49], [51], and 3 SR-MAs (14.3%) were low quality [34], [35], [40](Table 2).

### Directions for Future CPG of LBP

Of the 7 CPGs that included acupuncture, 2 showed a similar recommendation compared with current research on SR-MAs [25], [30], but 5 CPGs were underestimated [3], [20], [23], [28], [29]. Only 1 CPG included manual therapy and showed effectiveness underestimation [28]. Similar to moxibustion, cupping therapy and herbal medicine were not discussed in current CPGs and thus could not be compared.

We moderately recommended acupuncture for chronic LBP with a 1+/A evidence level and recommendation grade. However, we inconclusively recommended acupuncture for (sub)acute LBP due to the current SR-MA evidence. We weakly recommended cupping therapy for both (sub)acute and chronic LBP with a 1−/B evidence level and recommendation grade. We weakly recommend manual therapy for both (sub)acute and chronic LBP with a 1−/B evidence level and recommendation grade. Moxibustion and herbal medicine were not applicable due to the lack of data available at this time (Table 3).

**Table 3:** Directions for future Clinical Practice Guideline of Low Back Pain.

	Database	Guideline & Year	Acupuncture		Moxibustion		Cupping Therapy		Manual Therapy		Herbal Medicine	
Current												
	NGC (USA)	NGC-8959 2012	–		–		–		–		–	
		NGC-8744 2011	–		–		–		–		–	
		NGC-8517 2011	U		–		–		U		–	
		NGC-8193 2010	–		–		–		–		–	
		NGC-8009 2010	–		–		–		–		–	
		NGC-7704.2009	E		–		–		–		–	
		NGC-7510 2009	–		–		–		–		–	
		NGC-7428 2009	–		–		–		–		–	
		NGC-6456 2007	U		–		–		–		–	
		NGC-5968 2007	U		–		–		–		–	
	NICE (UK)	CG-88 2009	U		–		–		–		–	
	G-I-N(International)	Prodigy (UK)2009	U		–		–		–		–	
	MINDS(Japan)	CPG for the managementof LBP 2012	E		–		–		–		–	
Future												
	AuthorsRecommendation	Condition of LBP	Level of Evidence	Grade of Recommendation	Level of Evidence	Grade of Recommendation	Level of Evidence	Grade of Recommendation	Level of Evidence	Grade of Recommendation	Level of Evidence	Grade of Recommendation
		(Sub)acute	1−	B	–	–	1−	B	1−	B	–	–
		Chronic	1+	A	–	–	1−	B	1−	B	–	–
			Moderate recommendation for chronic LBP/Inconclusive for (Sub)acute LBP		Do not know		Weak recommendation for both (Sub)acute and chronic LBP		Weak recommendation for both (Sub)acute and chronic LBP		Do not know	

Abbreviations: U, underestimated; E, enough.

–:There were no available data, and assessments were not applicable.

## Discussion

The main aim of our review was to analyze TMs in East-Asian countries (China, Korea, and Japan) in the current CPGs for LBP. The results showed that TMs in East-Asian countries were not sufficiently included in current CPGs.

Notably, moxibustion, cupping therapy, and herbal medicine are not mentioned in current CPGs. The lack of eligible RCTs and the aggregation of SR of moxibustion for LBP might be the primary causes of this lack of inclusion. This omission leads to a lack of evidence for the CPG. The use of moxibustion has become more common; 67% of Korean Oriental medical doctors have used moxibustion [53]. Additionally, 40% of health care in China is currently based on traditional Chinese medicinal approaches that include moxibustion [54]. The adverse effects and difficulties of placebo moxibustion that are reported in the literature [55] may have emerged due to the limited moxibustion studies.

The relevant studies on cupping therapy were of poor quality [56], which might lead to a lack of inclusion in current CPGs. However, the only SR showed a positive effect for both sub(acute) and chronic LBP.

The heterogeneity of herbal medicine products may be a considerable problem. The various types of preparation and the amount of chemical constituents per dose influence the pharmacokinetics and relative efficacy of the herbal medicine [57]. These differences may make it difficult to conduct a high-quality study.

Some acupuncture recommendations had both favorable and unfavorable conclusions. Of all of the studies, 7 CPGs (54%) mentioned acupuncture, but only 1 study recommended acupuncture for chronic LBP without use limitations. The other studies recommended acupuncture for limited treatment sessions or did not recommend acupuncture. These results demonstrate a gap with the results of current SR-MAs. Negative or insufficient effects in SR-MAs were dominant for (sub)acute LBP [31]–[33], [35], [41]. However, the positive effects were dominant for chronic LBP [35], [36], [38], [39], [41]–[44]. Similar results were reported for the evidence-based medicine approach to LBP [58]. Therefore, we conclude that the recommendations for (sub)acute LBP seem appropriate, and the recommendations for chronic LBP are underestimated.

Regarding manual therapy, 1 CPG mentioned acupressure but did not recommend acupressure for both (sub)acute and chronic LBP. However, we found some gaps in SR-MAs. There were 5 SR-MAs with a positive result [47]–[51] (1 on Chuna [51] and 4 on acupressure or acupuncture massage [47]–[50]) and 1 insufficient SR on Shiatsu [49]. Additionally, 1 related SR-MA that was not included in this study supported the possibility of Tuina-integrated treatment t for LBP [59]. Thus, we find that the current CPGs underestimate the effectiveness of TM manual therapy for LBP.

The overall evidence available was usually published in the US and European countries, Thus, a lack of familiarity with East-Asian TM may influence the lack of interventions. These problems may explain the underpowered evidence for TM.

One important thing to consider is the need for more objective methods in TM practice. In contradistinction to classical approaches in Eastern medicine, where the methodology is much more concrete, TM is mainly considered an “art”. This understanding complicates an objective study of the results.

We also conducted a quality assessment that included CPGs and SR-MAs. The AGREE assessment showed that the quality of included CPGs was acceptable. The average scores of 5 domains (with the exception of 1 domain) were greater than 60%, and the mean score in the overall assessment was 4±1 [range: 3–6], indicating a moderate quality of CPG (Table 1). The domains of applicability obtained the lowest score, suggesting that more attention should be paid to quality enhancement during CPG development (Table S1).

The SR-MAs were assessed using the AMSTAR instrument [19]. Although the item “Was a priori design provided?” received the lowest score, the overall scores were quite high. The total mean score showed moderate quality of 6.59 [range: 2–10] for included SR-MAs (Table S2). Therefore, future authors should conduct an a priori design to ensure better study quality.

The strength of our study is its successful completion of the first review of TM in current CPGs for LBP. Previous CPGs of Traditional Chinese Medicine [60] did not focus on specific diseases or make further suggestions to address the lack of evidence. To prevent this bias, we attempted to determine whether current TM interventions were adequately included in current, rigorous CPGs of LBP. Thus, we searched current available CPGs and SR-MAs with systematic search methods and assessed CPG and SR-MA quality. In this study, we reanalyzed the evidence level and grade of recommendation of SR-MAs and aimed to identify directions for future research via a CPG-related expert consensus.

Several study limitations should be considered. Despite our best efforts to retrieve all CPGs and SR-MAs on this subject, we are not convinced that our search was inclusive. Notably, the definition of manual therapy categories in Oriental medicine is a considerable problem. Because we selected the subjects for TM in East-Asian countries, there is a potential question regarding whether the 5 types of intervention represent all TM interventions. To address this problem, we selected the interventions that consider the use of traditional Chinese medicine [61]. Although we suggested recommendation reassessments, we did not follow the entire procedure involved in crafting CPGs [62]. Instead, we made decisions via the expert consensus method. Therefore, biased conclusions are possible.

To address these weaknesses, we suggest important recommendations for future research in this area. First, high-quality RCTs were not conducted despite the use of TM, and there is a remarkable lack of studies on moxibustion, cupping therapy, Tuina (or Chuna), and herbal medicine, which deserve increased interest and further study. Second, a broader scope of TM interventions should be searched in further studies, and accurate recommendations for TM interventions should be drawn via proper procedures by larger organizations or teams. The increasing TM evidence should be included in the process of updating CPGs, and TM interventions based on LBP CPGs should be developed in collaboration with TM experts.

## Conclusion

Although interest in and use of TM is increasing, the CPGs identified did not fully reflect the TM interventions in East-Asian countries. In particular, acupuncture, cupping therapy, and manual therapy were underestimated or not mentioned despite their current evidence. The current evidence on acupuncture for chronic LBP and evidence on cupping and manual therapy for both (sub)acute and chronic LBP should be rigorously considered in the process of developing or updating the CPG system. However, a lack of evidence on moxibustion and herbal medicine prevented us from providing recommendations in these areas.

## Supporting Information

Table S1 **Assessment of Clinical Practice Guidelines (CPG) by AGREEII.**
(DOCX)

Table S2 **Assessment of Systematic Reviews by AMSTAR.**
(DOCX)

Checklist S1 **PRISMA checklist.**
(DOCX)
