# Supplementary material for: How Current Clinical Practice Guidelines for Low Back Pain Reflect Traditional Medicine in East Asian Countries: A Systematic Review of Clinical Practice Guidelines and Systematic Reviews
Source: PLoS One. 2014 Feb 5;9(2):e88027. doi: 10.1371/journal.pone.0088027 (PMC3914865; doi:10.1371/journal.pone.0088027)
Supplement: Table S1 — Assessment of Clinical Practice Guidelines (CPG) by AGREEII. (DOCX) [file pone.0088027.s001.docx]

| **Table S1. Assessment of Clinical Practice Guidelines (CPG) by AGREEⅡ.** | | | | | | | | |
| --- | --- | --- | --- | --- | --- | --- | --- | --- |
| **CPG**  **&**  **Year** | **Domain 1.**  **Scope and**  **Purpose** | | **Domain 2.**  **Stakeholder involvement** | **Domain 3.**  **Rigour of development** | **Domain 4.**  **Clarity of**  **presentation** | **Domain 5.**  **Applicability** | **Domain 6.**  **Editorial Independence** | **Overall Assessment** |
| CPG for the management of LBP  2012 | | 100 | 83 | 69 | 100 | 4 | 0 | 4/YWM |
| NGC-8959  2012 | | 100 | 100 | 75 | 100 | 100 | 100 | 6 / Y |
| NGC-8744  2011 | | 100 | 78 | 77 | 100 | 67 | 100 | 5 / YWM |
| NGC-8517  2011 | | 100 | 89 | 71 | 100 | 100 | 100 | 6 / Y |
| NGC-8193  2010 | | 50 | 56 | 46 | 89 | 17 | 75 | 3 / YWM |
| NGC-8009  2010 | | 33 | 56 | 52 | 72 | 25 | 58 | 3 / YWM |
| NGC-7704  2009 | | 39 | 50 | 73 | 50 | 46 | 50 | 3 / YWM |
| NGC-7510  2009 | | 78 | 61 | 83 | 100 | 0 | 0 | 3 / YWM |
| NGC-7428  2009 | | 72 | 67 | 81 | 67 | 42 | 100 | 4 / YWM |
| NGC-6456  2007 | | 61 | 78 | 60 | 67 | 79 | 100 | 4 / YWM |
| NGC-5968  2007 | | 50 | 50 | 52 | 39 | 71 | 33 | 3 / YWM |
| CG-88  2009 | | 100 | 89 | 85 | 83 | 46 | 58 | 5 / YWM |
| Prodigy  2009 | | 89 | 44 | 35 | 100 | 8 | 50 | 3 / YWM |
| Mean±SD | | 75±26 | 69±18 | 66±16 | 82±21 | 47±35 | 63±37 | 4±1 |

All AGREE II items are rated on the following 7-point scale: Score of 1 (Strongly Disagree)= There is no information that is relevant to the AGREE II item or the concept is very poorly reported.; Score of 7 (Strongly Agree)= quality of reporting is exceptional and the full criteria and considerations articulated in the User’s Manual were met.; Scores between 2 and 6= The reporting of the AGREE II item does not meet the full criteria or considerations. A score is assigned depending on the completeness and quality of reporting. Scores increase as more criteria are met and considerations addressed. We classified scores of 1 or 2 as low quality, scores of 3, 4 or 5 were moderate quality and 6 or 7 were high quality.; Domain scores are calculated by summing all the scores of the individual items in a domain and by scaling the total as a percentage of the maximum possible score for that domain. The scaled domain score will be: (Obtained score – Minimum possible) score / (Maximum possible score – Minimum possible score)*100; YMA: yes with modification; Y:yes
