# Supplementary material for: How Current Clinical Practice Guidelines for Low Back Pain Reflect Traditional Medicine in East Asian Countries: A Systematic Review of Clinical Practice Guidelines and Systematic Reviews
Source: PLoS One. 2014 Feb 5;9(2):e88027. doi: 10.1371/journal.pone.0088027 (PMC3914865; doi:10.1371/journal.pone.0088027)
Supplement: Table S2 — Assessment of Systematic Reviews by AMSTAR. (DOCX) [file pone.0088027.s002.docx]

| **Table S2. Assessment of Systematic Reviews by AMSTAR.** | | | | | | | | | | | | | |
| --- | --- | --- | --- | --- | --- | --- | --- | --- | --- | --- | --- | --- | --- |
| **Intervention** | **First author**  **& Year** | **Items of AMSTAR** | | | | | | | | | | | |
|  |  | **A** | **B** | **C** | **D** | **E** | **F** | **G** | **H** | **I** | **J** | **K** | **Total** |
| Acupuncture | Hutchinson 2012 | N | Y | N | N | N | Y | N | N | Y | N | Y | 4 |
|  | Furlan 2012 | N | Y | Y | Y | Y | Y | Y | Y | Y | Y | N | 9 |
|  | Lu 2011 | N | Y | Y | Y | N | Y | Y | Y | Y | N | Y | 8 |
|  | Trigkilidas. 2010 | N | N | Y | Y | N | N | N | N | NA | N | N | 2 |
|  | Rubinstein 2010 | N | Y | Y | Y | Y | Y | Y | Y | Y | Y | Y | 10 |
|  | Yuan 2009 | N | Y | Y | N | Y | Y | Y | Y | N | N | Y | 7 |
|  | McIntosh. 2008 | N | U | Y | N | N | Y | Y | Y | N | N | N | 4 |
|  | McIntosh. 2008 | N | U | Y | N | N | Y | Y | Y | N | N | N | 4 |
|  | Ammendolia 2008 | N | Y | Y | N | N | Y | Y | Y | U | N | N | 5 |
|  | Maurits 2005 | N | U | U | U | N | Y | Y | Y | N | N | N | 3 |
|  | Manheimer. 2005 | N | Y | Y | Y | Y | Y | Y | Y | Y | N | Y | 9 |
|  | Furlan 2005 | U | Y | Y | Y | N | Y | Y | Y | Y | Y | N | 8 |
|  | Henderson 2002 | N | N | N | N | Y | Y | N | N | NA | N | N | 2 |
|  | Ernst 2002 | N | Y | Y | Y | Y | Y | Y | Y | Y | Y | Y | 10 |
|  | Lesley 2000 | N | Y | Y | Y | N | Y | Y | Y | U | N | Y | 7 |
|  | Tulder 1999 | N | Y | Y | Y | Y | Y | Y | Y | Y | N | N | 8 |
| Cupping | Kim 2011 | N | Y | Y | Y | N | Y | Y | Y | Y | N | Y | 8 |
| Manual therapy | Moon 2012 | N | Y | Y | N | N | Y | Y | Y | N | N | Y | 6 |
|  | Kim 2012 | N | Y | Y | Y | Y | Y | Y | Y | Y | Y | Y | 10 |
|  | Robinson 2011 | N | Y | Y | U | N | Y | Y | Y | NA | N | Y | 6 |
|  | Furlan 2009 | Y | Y | Y | Y | Y | Y | Y | Y | Y | N | Y | 10 |
|  | Imamura. 2008 | N | Y | Y | N | N | Y | Y | Y | NA | N | N | 5 |
|  | Mean±SD | 0.05±0.21 | 0.77±0.43 | 0.86±0.35 | 0.55±0.51 | 0.41±0.50 | 0.95±0.21 | 0.86±0.35 | 0.86±0.35 | 0.50±0.51 | 0.23±0.43 | 0.55±0.51 | 6.59±2.65 |

Y=yes; N=no; U= unclear; NA= not applicable; Items of AMSTAR: A = Was an “a priori” design provided?, B = Were there duplicate study selection and data extraction?, C = Was a comprehensive literature search performed?, D = Was the status of publication (i.e., grey literature) used as an inclusion criterion?, E =  Was a list of studies (included and excluded) provided?, F = Were the characteristics of the included studies provided?, G = Was the scientific quality of the included studies assessed and documented?, H=Was the scientific quality of the included studies used appropriately in formulating conclusions?, I = Were the methods used to combine the findings of studies appropriate?, J = Was the likelihood of publication bias assessed? K = Were potential conflicts of interest included?.; Each item received a score of one if the specific criterion was met or zero if not reported, unclear, or not applicable. The total AMSTAR score was calculated by adding the average scores for all 11 items. We averaged item scores across guidelines. The item scores were classified such that 0-3 indicated low quality, 4-7 indicated moderate quality and 8-11 indicated high quality
